# Levels of State and Trait Anxiety in Patients Referred to Ophthalmology by Primary Care Clinicians: A Cross Sectional Study

**DOI:** 10.1371/journal.pone.0065708

**Published:** 2013-06-13

**Authors:** Christopher J. Davey, Clare Harley, David B. Elliott

**Affiliations:** 1 Bradford School of Optometry and Vision Science, Bradford, United Kingdom; 2 School of Healthcare, University of Leeds, Leeds, United Kingdom; Harvard Medical School, United States of America

## Abstract

**Purpose:**

There is a high level of over-referral from primary eye care leading to significant numbers of people without ocular pathology (false positives) being referred to secondary eye care. The present study used a psychometric instrument to determine whether there is a psychological burden on patients due to referral to secondary eye care, and used Rasch analysis to convert the data from an ordinal to an interval scale.

**Design:**

Cross sectional study.

**Participants and Controls:**

322 participants and 80 control participants.

**Methods:**

State (i.e. current) and trait (i.e. propensity to) anxiety were measured in a group of patients referred to a hospital eye department in the UK and in a control group who have had a sight test but were not referred. Response category analysis plus infit and outfit Rasch statistics and person separation indices were used to determine the usefulness of individual items and the response categories. Principal components analysis was used to determine dimensionality.

**Main Outcome Measure:**

Levels of state and trait anxiety measured using the State-Trait Anxiety Inventory.

**Results:**

State anxiety scores were significantly higher in the patients referred to secondary eye care than the controls (p<0.04), but similar for trait anxiety (p>0.1). Rasch analysis highlighted that the questionnaire results needed to be split into “anxiety-absent” and “anxiety-present” items for both state and trait anxiety, but both subscales showed the same profile of results between patients and controls.

**Conclusions:**

State anxiety was shown to be higher in patients referred to secondary eye care than the controls, and at similar levels to people with moderate to high perceived susceptibility to breast cancer. This suggests that referral from primary to secondary eye care can result in a significant psychological burden on some patients.

## Introduction

In most developed countries patients with eye disease are detected within primary care by physicians or optometrists and then referred to ophthalmology in secondary care. Under-referral would lead to patients with eye disease being missed (false negatives), so there may be a tendency for optometrists and physicians to refer if in doubt. The threat of litigation may increase this tendency. False positive referrals, i.e. the referral of patients without eye disease, are partly a consequence of case finding a disease of low prevalence (glaucoma [1]) as well as a consequence of over-referral. The level of false positive referral to secondary eye care centers can be high. For example the largest sized studies suggested false positive rates of 46% (N = 1,106) [2] or 48% (N = 2,505) [3] for suspect glaucoma referrals by optometrists and the proportion of false positives from the patients evaluated in the present study (N = 392; all eye disease types; N = 100 for glaucoma suspects) was evaluated elsewhere and found to be approximately 30% (Davey CJ, PhD thesis).

The psychological consequences of referrals (including false positive referrals) in ophthalmology are not known. Issues of wasted time and resources are acknowledged [4] but the impact of referrals on patients’ psychological wellbeing is yet to be explored. In other fields of research, false positive referrals have been shown to negatively affect patients. Systematic reviews of the effect on patients of mammography screening for cancer concluded that women experience significant anxiety in both the short term and the long term [5], [6]. Studies on screening for congenital hypothyroidism [7] and pre-natal screening for Down’s syndrome [8] both indicate increased psychological distress related to false positive screening results.

In this study, we assessed the levels of anxiety present in 322 patients referred to a UK hospital ophthalmology department using the State-Trait Anxiety Inventory (STAI) and compared this to data from 80 age-matched control patients from optometric practice and also normative data from the STAI manual [9]. The STAI was chosen as it allows differentiation of anxiety into state (i.e. current transient anxiety level) and trait anxiety (i.e. propensity for the patient to be anxious) and is a widely used assessment of anxiety [10]–[14]. The STAI uses traditional Likert scoring and provides ordinal data so Rasch analysis was used to convert the data into an interval scale and assess the usefulness of individual items [15]–[18]. In addition, principal components analysis was used to ensure that any scale or subscale we used in the analyses were providing unidimensional data. For an eye care population, Rasch analysis has only been previously performed on the 6-item STAI 19,20, where it was used to provide interval data and was found to be unidimensional, although it provided relatively poor patient separation as is common with instruments using a small number of items.

## Methods

### Ethics Statement

The study complied with the tenets of the Declaration of Helsinki and ethical approval was given by the Bradford NHS Research Ethics Committee (Reference 07/Q1202/41). Eligible participants (identified using the hospital booking system) were new patients who had an outpatient appointment booked at Bradford Royal Infirmary Eye Service between January 2008 and December 2008. All eligible patients (1,854 patients) were sent a covering letter, an information sheet, contact details of the research team and a coded STAI questionnaire. The covering letter asked the patient to read the information sheet and, if they consented to participate, to complete the questionnaire on the day of their appointment and hand the completed questionnaires to the doctor or nurse who examined them on the day of their appointment. This was accepted as implied written consent by the ethics committee as it meant that no patient identifiable data had to be sent via post. No children participated in the study.

### Inclusion and Exclusion Criteria

Inclusion criteria for the referred cohort included an initial referral from a GP or optometrist to the hospital eye service within the proposed testing schedule of the study, and aged over 16. Exclusion criteria included patients who were already hospital patients and had been called back for review or requiring further investigation.

### Measures

The STAI contains 40 items, 20 aimed at State anxiety (current level of anxiety), followed by 20 for Trait anxiety (propensity for the patient to be anxious). 21 of the items are anxiety-present items (e.g. “I feel nervous and restless”) and 19 are anxiety-absent items (e.g. “I feel pleasant”). Items are scored on a four point Likert scale, scored 1–4, with the anxiety-absent items being reverse scored.

### Procedure

The information was sent to arrive by post at least 24 hours in advance of their appointment at the hospital. If the patients read the information and subsequently consented to participate they were requested to bring the anonymized but coded questionnaires on the day of their appointment. The consenting patients were asked to complete the questionnaire, which should have taken about 10 minutes, while they were waiting for their appointment and if they wished to complete them at the hospital a private room was available. When the participants were called for their appointment they were asked to hand the completed questionnaires, in the sealed envelope provided, to the clinician to be passed on to the researcher. Identifying codes were used, to anonymize patients’ responses. Codes were cross-referenced at a later stage to unite questionnaire and patient demographic data.

### Control Group

In order to determine whether hospital patients had raised levels of psychological distress, the level of distress in a control group also had to be determined. The most suitable control group was patients that had an eye examination in primary care but had not been referred. Local optometric practices were approached via a Local Optical Committee meeting and invited to recruit patients on our behalf. Seven optometry practices agreed to participate. The optometrists asked all patients within the inclusion criteria (over 16 years of age and not needing referral to secondary eye care) if they would participate in the study and those who were interested were given information sheets and the questionnaires.

### Statistical Analysis

SPSS Statistics for Windows, Version 17.0 (Chicago: SPSS Inc.) was used to perform a Kolmogorov-Smirnov test for normality of the data distribution. Where appropriate, non-parametric statistical analyses were used to detect the significance of any differences between groups. Rasch analysis using Winsteps 3.66 was used to assess individual items in terms of their fit to the Rasch model using mean square fit statistics (infit and outfit). Items with fit statistics greater than 1 demonstrate more variation from the predicted model and if too high may be unreliable or measure a different trait to the rest of the scale. Conversely, items with fit statistics less than 1 lack variance from the model and if too low are too predictable meaning they may not help discriminate between participants. The present study identified misfitting items if their infit or outfit values were outside the range 0.7 to 1.3 [21]–[23]. Misfitting items were removed and the analysis run again to determine the effect it had on the participant discrimination (as measured by the Participant Separation Index, PSI). The distribution of responses to the categories of each item was assessed i.e. floor and ceiling effects, and Principal Components Analysis (PCA) of the residuals was performed to determine the dimensionality of the STAI.

## Results

322 (17% of those posted) STAI questionnaires were completed with up to two missed items by the hospital cohort, and 80 were completed by control participants. The respondents were similar in age and gender to the non-respondents with a mean age of 61 (SD 19) compared to 58 (SD 19) and with both having a gender mix of 54% female. Ethnicity information was not obtained until patients had consented; therefore these data were not available for the non-respondents. Age and gender mix were similar for the control cohort (Table 1), although the main cohort was slightly more ethnically diverse and included 27% of participants who had not self-specified their ethnicity compared to 8% in the control group.

**Table 1:** Demographic data for the main cohort and the control group.

Characteristic	Patients referred (n = 322)	Control (n = 80)
Mean age (years ± SD)	61±19	61±16
Gender: Female	170 (53%)	43 (53.75%)
Gender: Male	144 (45%)	30 (37.5%)
Gender: Unspecified	8 (2%)	7 (8.75%)
Ethnicity: White	188 (58%)	71 (89%)
Ethnicity: Asian	39 (12%)	2 (3%)
Ethnicity: Black	6 (2%)	1(1%)
Ethnicity: Not Stated	88 (27%)	6 (8%)
Ethnicity: Chinese	1 (<1%)	

White ethnicity included White (British), White (Irish) and White (other) and was predominantly White (British). Asian ethnicity included Asian (Indian), Asian (Pakistani), Asian (Bangladeshi) and Asian (other). Black ethnicity included Black (African), Black (Caribbean) and Black (other). SD, standard deviation.

### Rasch Analysis and Principal Components Analysis

**Figure 1:**
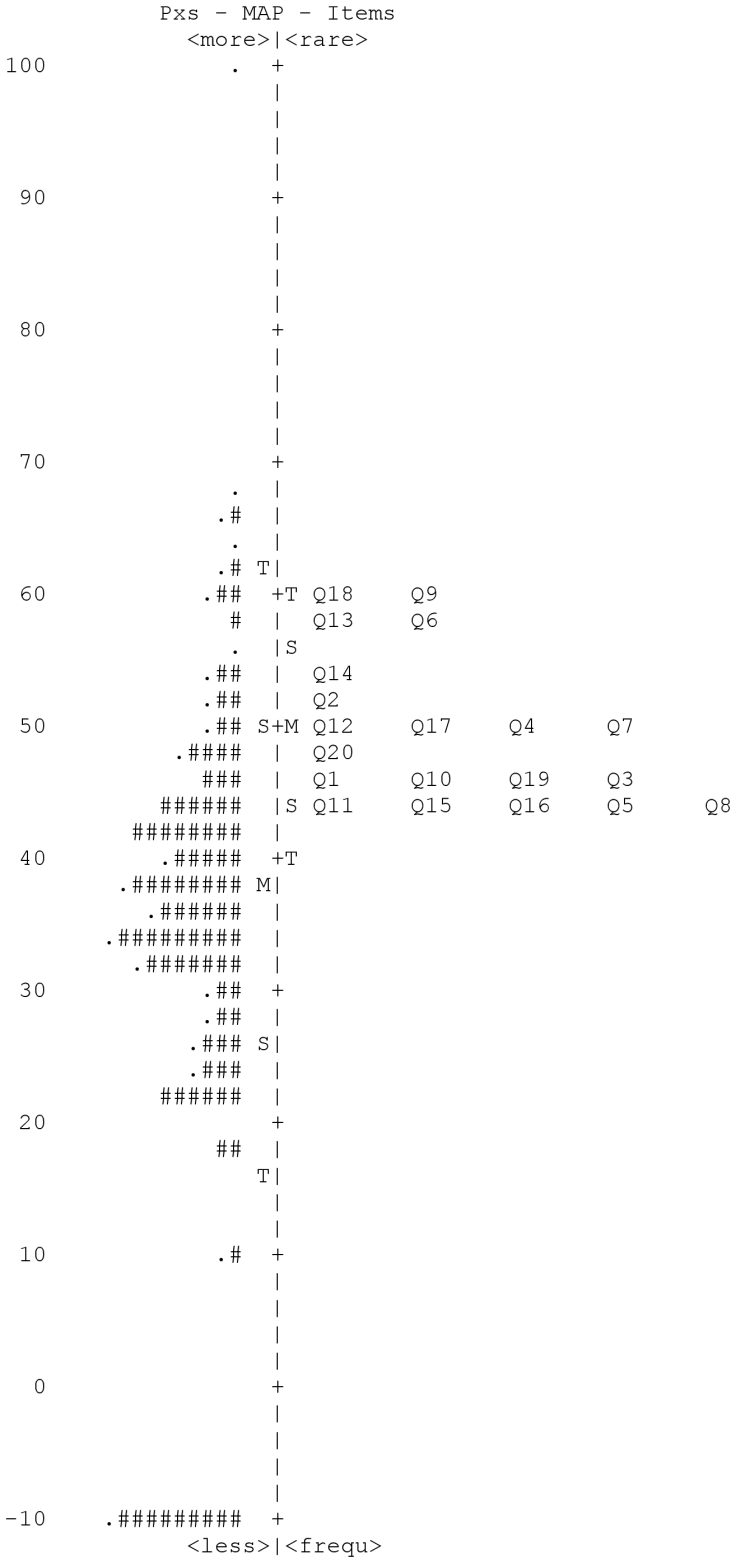
STAI-State Item-participant map for the hospital cohort. Each ‘#’ is 3 participants (Pxs).

The STAI-State item-person map containing participants from the main hospital cohort is shown in figure 1. A floor effect was present for STAI-State data with <10% of participants endorsing response category 4 for any item. Items 6, 9 and 18 had kurtosis values over the cutoff of 2, and items 3, 4, 6, 13, 14, and 15 had fit values outside the range 0.7–1.3, thus according to suggested guidelines for response scale reduction [24], [25], response categories 3 and 4 were combined. This improved the PSI from 2.71 to 2.75, improved the fit, skew and kurtosis values and reduced the difference between the participant mean and item mean from 12.3 to 6.8. STAI-Trait showed similar results, combining response categories 3 and 4 improved the PSI from 3.0 to 3.1, improved the fit, skew and kurtosis values, and reduced the difference between the participant mean and item mean from 11.5 to 4.2.

**Figure 2:**
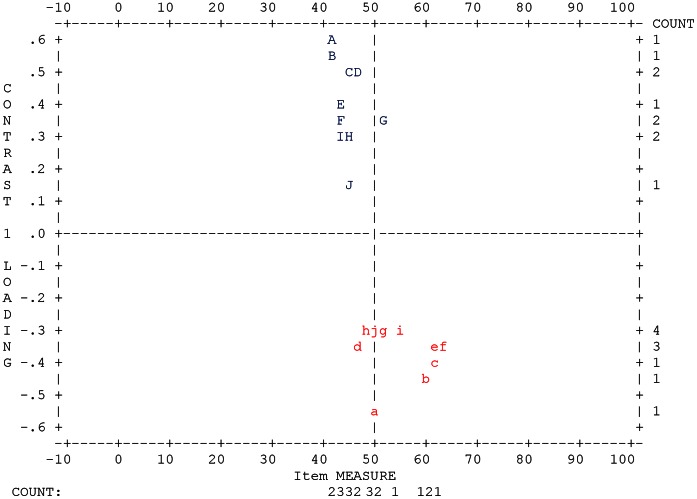
Standardized residual plot for Principal Components Analysis of STAI-S.

As stated in the methods, the STAI State and Trait anxiety subscales each have two types of item within them; anxiety absent questions and anxiety present questions, with the anxiety absent questions being reverse scored. This suggests the possibility that these anxiety-absent and anxiety-present factors within the State and Trait subscales could make the data multidimensional. This hypothesis was tested using Principal Components Analysis. The raw variance for STAI-State data explained by the measures after combination of response categories 3 and 4 was 47.8%, which is well below the 60% suggested as indicating unidimensionality and the eigenvalue of the first contrast was 3.0 suggesting that another significant dimension existed within the data. The 2^nd^ contrast had an eigenvalue of 1.7 indicating that there was not a third significant factor. The standardized residual data plot (Figure 2 and Table 2) showed a clear differentiation into two groups of data and matched the split of the items into state anxiety-present and state anxiety-absent factors. Despite both contributing towards the same construct, these two factors are clearly separate, therefore the items were split into two subscales and re-analyzed. Separate PCA for STAI-State anxiety-absent and STAI-State anxiety-present items suggested that separately the data were unidimensional (eigenvalues of the first contrast of 1.80 and 1.60 respectively). PCA for the STAI-Trait data showed very similar findings so these data were also separated into STAI-Trait anxiety absent and STAI-Trait anxiety present subscales.

**Table 2:** Letter to item number conversion key for figure 2.

Item	1	2	3	4	5	6	7	8	9	10	11	12	13	14	15	16	17	18	19	20
	J	G	d	g	F	c	j	E	f	C	I	a	b	i	B	A	h	e	H	D

Rasch fit statistics (Tables 3, 4, 5, 6) improved after separation into anxiety absent and anxiety present subscales, further supporting the lack of unidimensionality of the full scales. The STAI-State subscales showed no misfitting anxiety absent items (Table 3), but anxiety present item 14 had an outfit value of 1.39 (Table 4), although removal resulted in a decrease in PSI (from 1.69 to 1.61). Similarly for STAI-Trait subscales (Tables 5 and 6), misfit was found for anxiety absent item 34 (infit 1.48, outfit 1.69) and anxiety present item 24 (infit 1.56, outfit 1.64) but when removed both resulted in unacceptable reductions in PSI (from 2.04 to 1.96 for anxiety absent, and 2.31 to 2.28 for anxiety present). All items for all subscales were therefore retained to maximize participant discrimination. No significant differential item functioning was exhibited for any item of any subscale for age or gender (Bonferroni corrected t test) [26].

**Table 3:** Fit statistics for the STAI-State anxiety absent subscale.

Item	Infit	Outfit
1	1.1	1.2
2	0.9	0.9
5	1.0	1.0
8	1.1	1.2
10	0.9	0.9
11	1.2	1.3
15	0.7	0.7
16	0.9	0.9
19	1.2	1.2
20	1.0	0.9

**Table 4:** Fit statistics for the STAI-State anxiety present subscale.

Item	Infit	Outfit
3	1.0	1.2
4	1.0	1.0
6	1.0	0.9
7	1.0	1.0
9	1.0	1.0
12	0.8	0.8
13	0.9	1.0
14	1.2	1.4
17	1.0	1.0
18	0.9	0.9

**Table 5:** Fit statistics for the STAI-Trait anxiety absent subscale.

Item	Infit	Outfit
21	1.1	1.3
23	0.9	0.9
26	1.3	1.5
27	0.9	0.8
30	0.8	0.8
33	0.9	0.8
34	1.5	1.7
36	0.8	0.7
39	0.9	0.9

**Table 6:** Fit statistics for the STAI-Trait anxiety present subscale.

Item	Infit	Outfit
22	0.8	0.8
24	1.6	1.6
25	0.9	0.8
28	0.9	0.9
29	0.9	1.0
31	1.0	1.1
32	1.1	1.1
35	0.9	0.9
37	0.8	0.8
38	1.1	1.2
40	0.8	0.8

### Kolmogorov-Smirnov and Significance Testing

Kolmogorov-Smirnov testing showed the data to not be normally distributed (p<0.001), and median and inter-quartile range (IQR) data of STAI-State (Anxiety-Absent and Anxiety-Present) and STAI-Trait (Anxiety-Absent and Anxiety-Present) are shown in Table 7. Mann-Whitney U-tests showed that STAI-State data were significantly higher in referred patients compared to controls (Anxiety-Absent, p = 0.039; Anxiety-Present, p = 0.01). However, STAI-Trait data from referred patients and controls were similar (Anxiety-Absent, p = 0.16; Anxiety-Present, p = 0.103).

**Table 7:** Median item scores and Inter Quartile Ranges for Rasch-scored STAI-State and STAI-Trait, anxiety present and anxiety absent subscales.

	Control	Referred patients
STAI-State AA	39.7 (IQR 28.8–53.6)	44.8 (IQR 34.6–59.6)
STAI-State AP	32.9 (IQR 28.8–44.1)	37.2 (IQR 30.9–47.8)
STAI-Trait AA	44.8 (IQR 33.9–58.0)	50.2 (IQR 36.6–63.7)
STAI-Trait AP	37.2 (IQR 29.1–49.3)	40.7 (IQR 40.7–31.4)

AA, anxiety absent. AP, anxiety present. IQR, inter quartile range.

Because some participants did not complete any of the STAI-Trait items as they failed to turn over the last page of the questionnaire, and a few did not complete STAI-State but completed STAI-Trait, there were different numbers of respondents for each sub-scale (STAI-State n = 318, STAI-Trait n = 280). However, re-running the above analyses using only data from participants who completed both subscales (N = 276) found no differences to the results described above.

## Discussion

The STAI-State and STAI-Trait subscales were not unidimensional, but split into well-established and logical subscales with PCA. Both state and trait scales of the STAI showed good discriminative ability (PSI>2.0) and for both anxiety absent and present item subscales, apart from STAI-State Anxiety Present items which only achieved a maximum PSI of 1.69.

The PCA assessment of unidimensionality for both state and trait scales that showed two factors within the data agrees with the original author’s two factor model for anxiety present and anxiety absent items [9]. Multiple studies have since agreed that higher scores are provided by respondents for anxiety absent items such as “I feel…calm, at ease, satisfied, comfortable etc” compared to anxiety present items such as “I feel….strained, upset, frightened, jittery etc” [9], [27], [28]. This is because confirming the presence of anxiety is not psychologically equivalent to not confirming the presence of calmness. This has been confirmed as providing multidimensional data using PCA in this study.

Analysis of STAI-Trait (both anxiety-absent and anxiety-present item subsets) using Rasch-analysed data showed there was no significant difference in trait anxiety between the control cohort and the cohort that had been referred to secondary eye care (p>0.1). This means that the main cohort were not significantly more prone to being anxious (ie. Trait anxiety) than the control group. Analysis of STAI-State showed that levels of state anxiety, ie. how anxious the patient is “right there and then”, were significantly higher in the patients who had been referred to the hospital. This was true for both anxiety absent (p = 0.039) and anxiety present data (p = 0.01). This indicates that when some patients are referred to secondary eye care there may be a psychological burden, which is a similar finding to other areas of healthcare such as dentistry, oncology or screening for congenital syndromes [7], [8], [29]–[33]. State anxiety is highly reactive to context and environment, and as the main cohort completed their questionnaires whilst sat in a waiting room which contrasts to the controls who completed it at home, it is the whole experience of referral to the hospital which is being evaluated not just the anticipatory anxiety of being referred.

To determine whether the level of State anxiety in the referred ophthalmology patients was clinically significant it was not possible to use Rasch analyzed data as all previous pertinent studies had used traditional Likert scoring. We therefore calculated the Likert scores for the patients referred to secondary eye care (mean, SD: 35.6±12.7) and the control group (32.0±11.4). Compared to data from a breast cancer screening study [34], the control group scores were similar to those with low perceived susceptibility to breast cancer (∼31.5) and the referred patients’ scores were similar to patients with moderate to high perceived susceptibility to breast cancer (moderate ∼34, high ∼37). This suggests that the level of state anxiety measured in the referred ophthalmology patients was clinically significant as well as statistically significant. The mean score of 35.5 for STAI-State in the hospital patients aged 60–69 (n = 66) is also above the 95% confidence limits for normative working adult data for the 60–69 age group of 34.6 (mean ∼32.2) from the STAI manual [9].

Limitations of the study were that the number of control subjects was relatively small (N = 80 compared to referred patient N of 322) and the participants in both groups self selected which may introduce a self selection bias. The timescale of the effect on State anxiety was not investigated therefore we do not know whether anxiety levels return to normal if the clinician has indicated that the patient has healthy eyes and good vision, and this needs further research. However, raised anxiety in patients with false positive results in breast cancer screening remains to a lesser extent in the long term [5], [35] and may even reduce attendance at future screenings [5].

In summary, this research has demonstrated that referral to secondary eye care can raise anxiety to potentially ‘clinically significant’ levels. This should be considered as part of the decision of whether and how to screen for diseases, such as chronic open angle glaucoma, in primary eye care that are relatively rare [36] and where the potential for a large number of false positive referrals is high [2], [3]. It should be noted that any efforts to reduce levels of false positive referral need to take in to consideration the risk of significantly raising false negatives. Steps should be taken at the point of referral as well as within secondary care to acknowledge and reduce potential increased anxiety.
